# Sodium valproate increases activity of the sirtuin pathway resulting in beneficial effects for spinocerebellar ataxia-3 in vivo

**DOI:** 10.1186/s13041-021-00839-x

**Published:** 2021-08-20

**Authors:** Maxinne Watchon, Luan Luu, Katherine J. Robinson, Kristy C. Yuan, Alana De Luca, Hannah J. Suddull, Madelaine C. Tym, Gilles J. Guillemin, Nicholas J. Cole, Garth A. Nicholson, Roger S. Chung, Albert Lee, Angela S. Laird

**Affiliations:** 1 Centre for Motor Neuron Disease Research, Department of Biomedical Sciences, Faculty of Medicine, Health and Human Sciences, Macquarie University, Level 1, 2 Technology Place, Sydney, NSW 2109 Australia; 2 ANZAC Research Institute, Concord Repatriation Hospital, Concord, NSW Australia

**Keywords:** Machado−Joseph disease, Spinocerebellar ataxia−3, Zebrafish, Neurodegeneration, Sodium valproate, Valproic acid, Polyglutamine

## Abstract

**Abstract:**

Machado-Joseph disease (MJD, also known as spinocerebellar ataxia type 3) is a fatal neurodegenerative disease that impairs control and coordination of movement. Here we tested whether treatment with the histone deacetylase inhibitor sodium valproate (valproate) prevented a movement phenotype that develops in larvae of a transgenic zebrafish model of the disease. We found that treatment with valproate improved the swimming of the MJD zebrafish, affected levels of acetylated histones 3 and 4, but also increased expression of polyglutamine expanded human ataxin-3. Proteomic analysis of protein lysates generated from the treated and untreated MJD zebrafish also predicted that valproate treatment had activated the sirtuin longevity signaling pathway and this was confirmed by findings of increased SIRT1 protein levels and sirtuin activity in valproate treated MJD zebrafish and HEK293 cells expressing ataxin-3 84Q, respectively. Treatment with resveratrol (another compound known to activate the sirtuin pathway), also improved swimming in the MJD zebrafish. Co-treatment with valproate alongside EX527, a SIRT1 activity inhibitor, prevented induction of autophagy by valproate and the beneficial effects of valproate on the movement in the MJD zebrafish, supporting that they were both dependent on sirtuin activity. These findings provide the first evidence of sodium valproate inducing activation of the sirtuin pathway. Further, they indicate that drugs that target the sirtuin pathway, including sodium valproate and resveratrol, warrant further investigation for the treatment of MJD and related neurodegenerative diseases.

**Graphical abstract:**

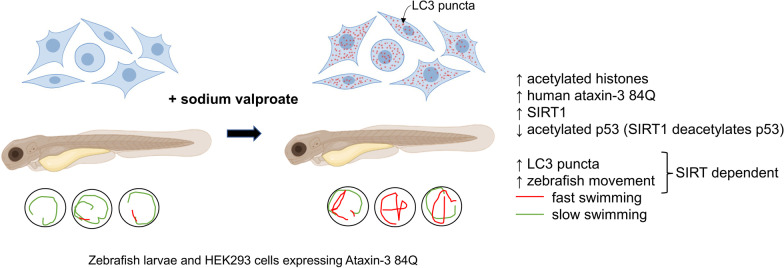

**Supplementary Information:**

The online version contains supplementary material available at 10.1186/s13041-021-00839-x.

## Background

Machado-Joseph disease (MJD), also known as spinocerebellar ataxia type 3, is a fatal neurodegenerative disease characterized by clinical symptoms including ataxia, dystonia, rigidity, muscle atrophy, and visual and speech disorder [10, 33, 36]. MJD is the most common of the hereditary ataxias found throughout the world (21–28% of autosomal-dominant ataxia) [12, 35, 38], with a high prevalence within the Azores of Portugal [1] and Indigenous communities of north east Arnhem Land in Australia [5].

MJD is caused by inheritance of an expanded CAG repeat region within the *ATXN3/MJD1* gene on chromosome 14 [29, 41]. In healthy subjects the *ATXN3* gene contains a short CAG trinucleotide repeat region containing 12–40 CAG repeats, but presence of over 40 CAG repeats in this region leads to MJD [2, 27, 28]. This expanded CAG repeat within the *ATXN3* gene results in presence of a long polyglutamine (polyQ) tract towards the C-terminus of the ataxin-3 protein [27, 28].

The ataxin-3 protein is known to function as a deubiquitinating enzyme (DUB) and can also regulate transcription through interactions with other transcriptional coactivators (histone acetyltransferases; HAT) [23]. Studies in models of MJD have demonstrated an interaction between human ataxin-3 (wild-type or mutant) and transcriptional repressor histone deactylase-3 (HDAC; class I HDAC), further highlighting a role for ataxin-3 in regulating transcription [14, 15]. Several animal models expressing human ataxin-3 containing an expanded polyQ region have shown hypoacetylation of histones 3 and 4 [8, 9, 24, 50] and increased HAT inhibition, suggesting that the expansion of the polyQ tract within the ataxin-3 protein may enhance HDAC activity [9, 23].

Whilst there is currently no effective treatment for MJD, a range of recent studies have indicated therapeutic candidates with potential benefit. One candidate that has been trialed in vitro*, *in vivo and in MJD patients is the class I and IIa HDAC inhibitor sodium valproate (valproate, or valproic acid) [13, 22, 24, 50]. Similarly, divalproex sodium (comprised of a combination of sodium valproate and valproic acid together) attenuates cytotoxicity in cellular models of MJD [45, 46].

Sodium valproate has FDA approval for the treatment of epilepsy and bipolar disorder, where it is thought to have a beneficial effect through blocking voltage gated sodium channels and increasing γ-aminobutyric acid (GABA) neurotransmission, respectively [7, 26]. Sodium valproate has also been investigated for a range of other neurodegenerative diseases, with clinical trials occurring in Huntington’s disease, Alzheimer’s disease, amyotrophic lateral sclerosis and MJD [16, 22, 32, 37]. Whilst multiple studies have demonstrated neuroprotective effects of valproate treatment, the mode-of-action of this neuroprotective effect is still poorly understood.

To gain a greater understanding of the potential benefits of treatment with valproate, we have trialed its treatment on our transgenic zebrafish model of MJD that express enhanced green fluorescent protein (EGFP)-tagged human ataxin-3 protein containing either an expanded polyQ tract (84Q, representing disease causing) or a short, wild-type, polyQ tract (23Q), under a neuronal promoter (HuC/elavl3) [47]. This model is very suitable to rapid treatment testing studies because treatments can be tested simply through their addition to the water the larvae swim in, and motor phenotypes develop rapidly (by 6 days post-fertilisation) [47]. Following our treatment study, we then carried out label-free proteomics, immunoblot analysis and microscopy to determine the potential mechanisms and cellular pathways by which valproate treatment improved movement in the transgenic MJD zebrafish, to identify a pathway for future investigation for the treatment of MJD.

## Results

### Transgenic MJD zebrafish carrying expanded polyQ length exhibit altered histone acetylation and sodium valproate can increase histone acetylation whilst improving motor function

**Fig. 1:**
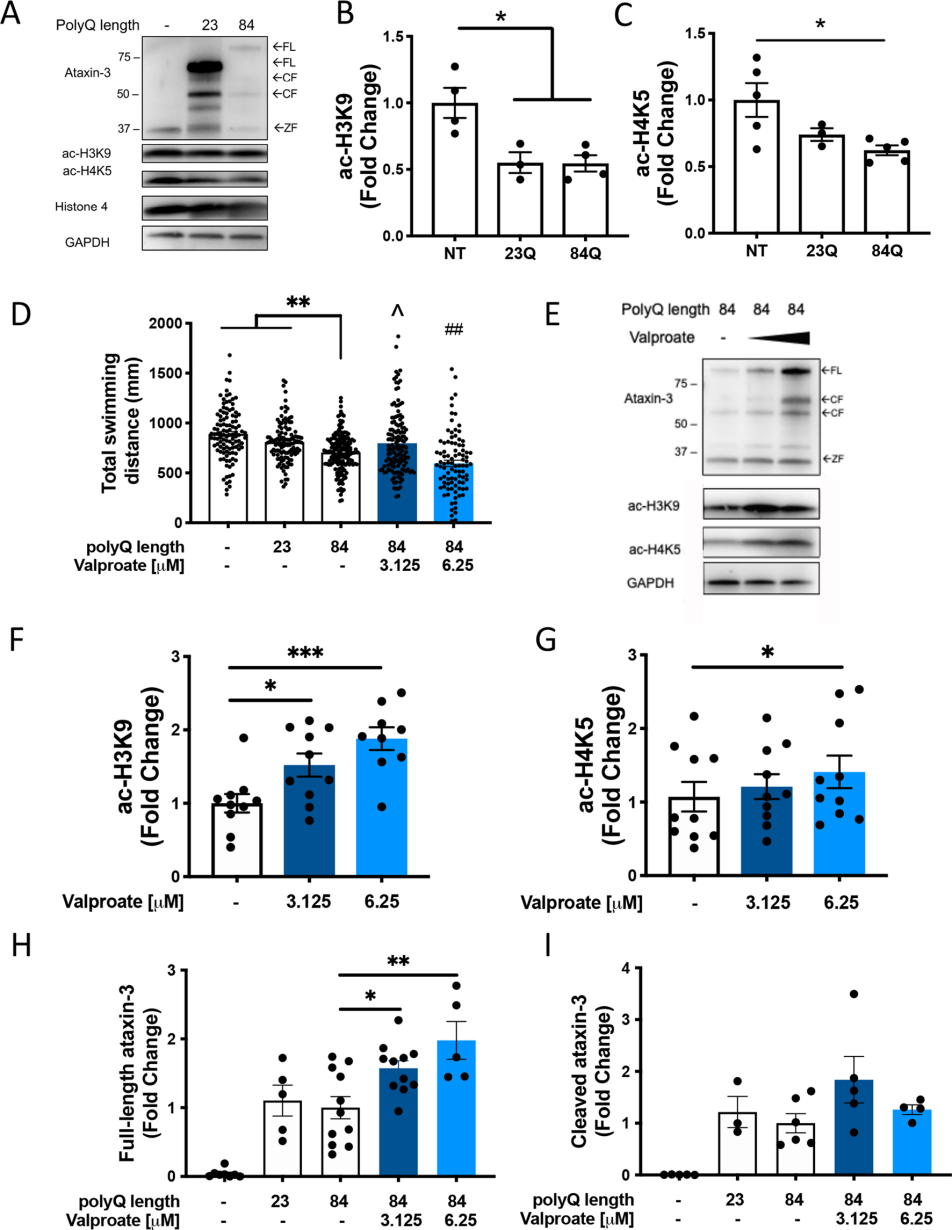
Levels of acetylated histones 3 and 4 are decreased in MJD zebrafish and sodium valproate increases levels of acetylated histones and locomotion in transgenic MJD zebrafish. **A** Immunoblots of 6-day-old transgenic MJD zebrafish expressed human ataxin-3 with full-length (FL) ataxin-3, cleaved (CF) ataxin-3 and endogenous zebrafish ataxin-3 (ZF). Transgenic MJD zebrafish also have decreased levels of acetylated histone 3 (ac-H3K9) and 4 (ac-H4K5) with no differences in histone 4. **B** Quantification of ac-H3K9 showed wild-type and mutant ataxin-3 larvae with significantly lower levels of ac-H3K9 compared to the non-transgenic control (*p < 0.022, n = 3–5). **C** Densitometric analysis of the amount of ac-H4K5 revealed a significant decrease in ac-H4K5 in the mutant ataxin-3 zebrafish compared to the non-transgenic control (*p = 0.026, n = 3–5). **D** Treatment of MJD zebrafish with low-dose sodium valproate (valproate; 3.125 µM) increased the distance travelled during movement tracking to control treated EGFP-Ataxin-3 84Q (^*p* = 0.019, n = 93–161). By contrast, high dose valproate (6.25 µM) decreased the distance travelled compared to EGFP-Ataxin-3 84Q, ##; *p* = 0.008. **E** Western blotting of valproate treated EGFP-Ataxin-3 84Q larvae resulted in increased levels of full-length human ataxin-3, ac-H4K5 and ac-H3K9. **F** Quantification of ac-H3K9 levels revealed valproate treatment increased ac-H3K9 in a dose dependent manner (*p = 0.004 and ***p = 0.003; n = 9). **G** Quantification of ac-H4K5 showed an increase with 6.25 µM valproate treatment (*p = 0.043, n = 10). **H** Quantification of human FL ataxin-3 showed significantly increased levels with both concentrations of valproate treatment compared to vehicle treated mutant ataxin-3 fish (*p = 0.0495, **p = 0.002, n = 5–11). **I** Quantification of human CF ataxin-3 relative to loading control showed no changes after treatment with sodium valproate (n = 3–6). Data represent mean ± SEM. Statistical analysis used in this figure were paired and unpaired one-way ANOVA with Tukey post-hoc analysis

Immunoblot analysis confirmed that the transgenic MJD zebrafish expressed full-length human ataxin-3 protein of appropriate sizes (72 kDa and 84 kDa for EGFP-Ataxin-3 23Q and 84Q, respectively), as well as endogenous zebrafish ataxin-3 (34 kDa) (Fig. 1A). Probing the immunoblots for acetylated histone 3 (ac-H3K9) and histone 4 (ac-H4K5) revealed a decrease in both with increasing ataxin-3 polyQ length (Fig. 1A), with significantly decreased levels of both ac-H3K9 and ac-H4K5 in EGFP-Ataxin-3 84Q larvae compared to non-transgenic animals (Fig. 1B-C).

We treated transgenic zebrafish with two doses of the HDAC inhibitor (3.125 μM and 6.25 μM) from 1–6 dpf and then performed motor behaviour testing and whole-body protein extraction for immunoblotting analysis. EGFP-Ataxin-3 84Q larvae swam shorter distances than both non-transgenic and EGFP-Ataxin-3 23Q controls (Fig. 1D), as described previously [47]. The impaired swimming of EGFP-Ataxin-3 84Q larvae was rescued by treatment with low-dose valproate (3.125 μM), resulting in increased swimming distance. A higher concentration of valproate (6.25 μM) did not improve swimming and in fact worsened it, suggesting that this higher drug concentration is reaching toxic levels. Accordingly, we identified an increased rate of morphological abnormalities in animals treated with the higher concentration (Additional file 1: A, B). For this reason, only low-dose 3.125 μM valproate treatment was explored further. Treating non-transgenic zebrafish with low-dose valproate from 1–6 dpf produced no changes in motor behavior compared to the vehicle control. This suggests that the rescue to motor impairment by valproate is specific to the expression of mutant ataxin-3.

Immunoblot analysis of whole-body lysates extracted from the various groups revealed that valproate treatment increased the levels of ac-H3K9 and ac-H4K5 (Fig. 1E). As it is known that sodium valproate is an inhibitor of histone deacetylases, valproate treatment produced a dose dependent increase in ac-H3K9 levels compared to that present in EGFP-Ataxin-3 84Q zebrafish receiving vehicle treatment (Fig. 1F). High dose valproate resulted in increased levels of ac-H3K9 compared to non-transgenic and EGFP-Ataxin-3 23Q controls (data not shown). High dose valproate also produced an increase in levels of ac-H4K5 (Fig. 1G). We also identified that valproate treatment produced a dose dependent increase in the amount of full-length human ataxin-3 present compared to in vehicle treated EGFP-Ataxin-3 84Q zebrafish (up to nearly twofold for high dose valproate; Fig. 1H). Despite this, the amount of cleaved human ataxin-3 (relative to loading control) did not differ between the valproate treated EGFP-Ataxin-3 84Q groups (Fig. 1I).

**Fig. 2:**
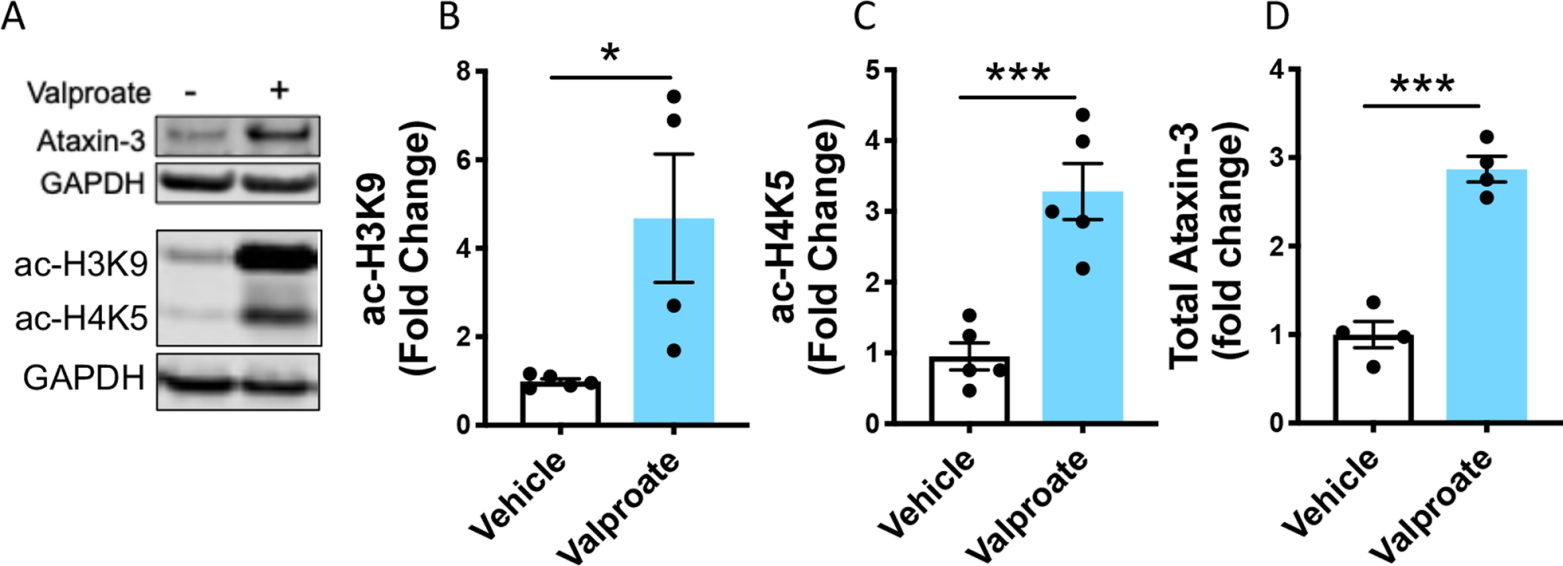
Sodium valproate treatment increased acetylated histones in HEK293 cells expressing human ataxin-3. **A** HEK293 cells expressing human ataxin-3 were treated with either sodium valproate (Valproate) or vehicle and protein lysates extracted from groups of larvae underwent immunoblot analysis for acetylated histones 3 and 4 and human ataxin-3. Quantification revealed that valproate treatment increased the amount of **B** acetylated histone 3 (ac-H3K9); **C** acetylated histone 4 (ac-H4K5); and **D** expressed human ataxin-3, compared to vehicle control treatment (*p = 0.023, ***p = 0.0007, ***p = 0.0001 respectively; n = 4–5 independent experiments). Data represents mean ± SEM and statistical analyses used were unpaired student *t*-tests

We then examined the effect of valproate treatment on HEK293 cells stably expressing human ataxin-3 84Q (without an EGFP-tag). Western blotting confirmed the expression of human ataxin-3 84Q (Fig. 2A). Valproate treatment (3 mM) produced significant increases in both ac-H3K9 and ac-H4K5 levels (Fig. 2B, C) and full-length human ataxin-3 levels, compared to vehicle controls (Fig. 2D), in a similar manner to the zebrafish findings.

### Unbiased label-free quantitative proteomics identifies various cellular pathways affected by sodium valproate

**Fig. 3:**
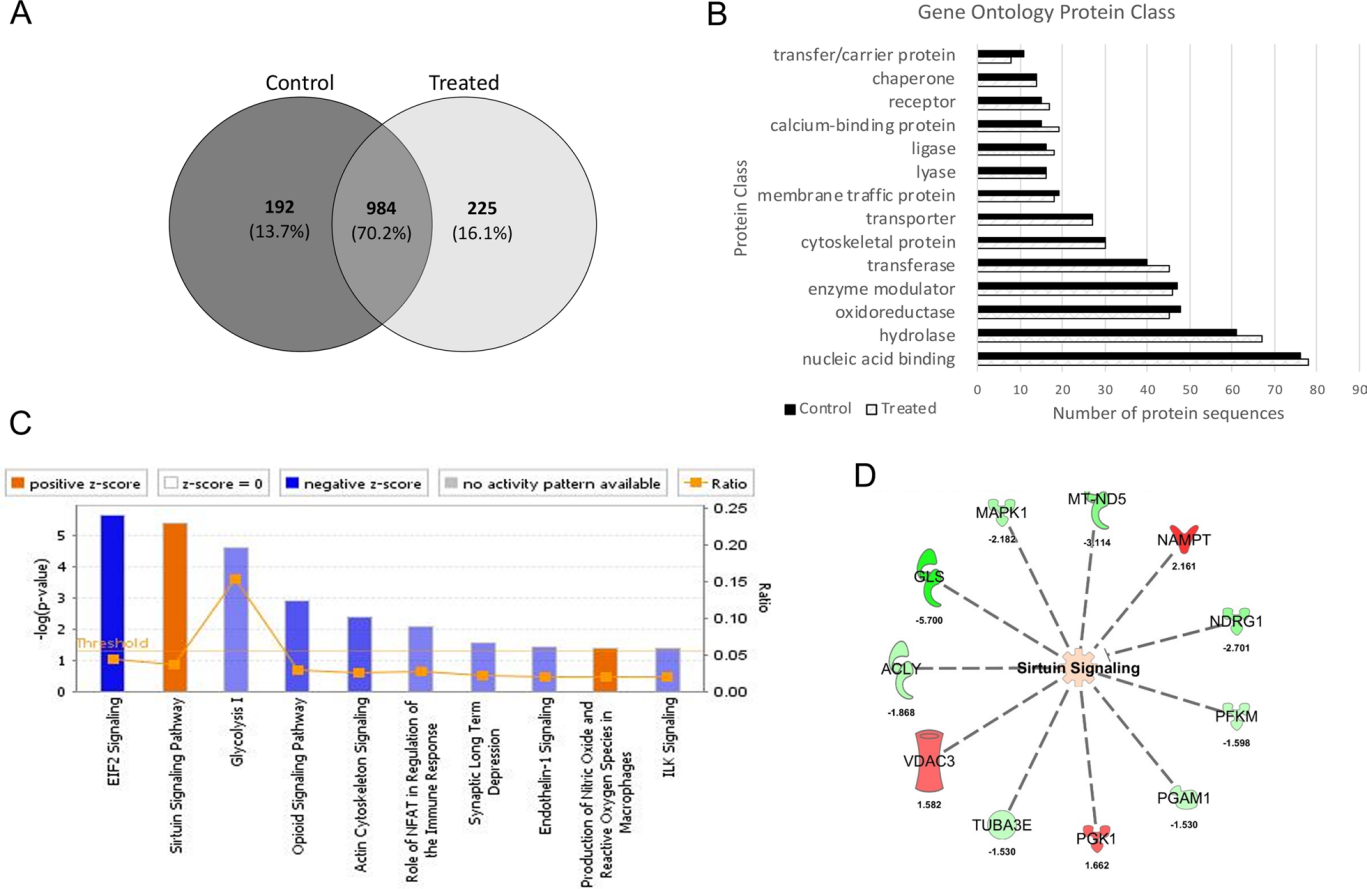
Label-free quantitative proteomics of EGFP-Ataxin-3 84Q transgenic zebrafish treated with vehicle and sodium valproate. **A** Triplicate analyses of vehicle and valproate treated transgenic zebrafish identified common and unique proteins. **B** Gene ontology (GO) annotation revealed small differences in the identification of proteins, with more proteins (9%) with hydrolase function, while less proteins (3%) were categorized to have carrier activity. **C** Ingenuity Pathway Analysis (IPA) predicted activation and inhibition of sirtuin signaling and EIF2 signaling pathways respectively upon valproate treatment of transgenic zebrafish expressing EGFP-Ataxin-3 84Q. Blue indicates IPA predicted inhibition and orange indicates predicted activation of categorised biological function and canonical pathways. **D** Predicted upregulation and downregulation of proteins associated with the sirtuin signaling pathway. Green indicates downregulation (0.67-fold) and red indicates upregulation (1.5-fold) of proteins in valproate treated EGFP-Ataxin-3 84Q zebrafish compared to the vehicle controls

Our finding of improved swimming following valproate treatment, together with increased expression of polyQ-expanded human ataxin-3, was surprising because we hypothesized that increased expression of polyQ-expanded human ataxin-3 would increase the severity of disease rather than improving motor function. This suggested that valproate treatment may also induce transcriptional activation of neuroprotective pathways that counteract the negative effects of increased levels of mutant ataxin-3. To identify the downstream molecular pathways that may be responsible for the improved movement of the MJD zebrafish we performed unbiased label-free quantitative proteomics to compare samples from transgenic zebrafish expressing EGFP-Ataxin-3 84Q treated with low-dose valproate (3.125 µM) or vehicle control. From triplicate analysis, we identified 1176 and 1209 proteins from vehicle and treated zebrafish respectively, with 984 (70.2%) proteins identified in common. We identified 192 (13.7%) and 225 (16.1%) unique proteins between the vehicle and valproate treated zebrafish lysates (Fig. 3A). Given the overlap (70.2%) of common proteins identified between control and treated conditions, we carried out gene ontology (GO) annotation of the unique protein lists between the two conditions to determine whether there were any differences in the protein classes and/or categories that would discriminate between the groups. We found that with valproate treatment, there were less proteins identified that were categorized as carrier proteins, whilst there were more proteins identified that were categorized as having transferase, hydrolase and calcium and nucleic acid binding function (Fig. 3B). Overall, there did not appear to be obvious differences in the protein classes that were identified between the vehicle and treated samples. Therefore, we employed Ingenuity Pathway Analysis (IPA) to interrogate the proteomics datasets and make predictions of the cellular pathways and functions that changed between treatment and control groups [6].

To determine differential (quantitative) protein expression between vehicle and valproate-treated transgenic zebrafish, we carried out label-free proteomics using normalized spectral abundance factors (NSAF) as described previously [53]. We identified approximately 61 downregulated (< 0.67-fold) and 86 upregulated (> 1.5-fold) proteins in valproate-treated zebrafish compared to the vehicle controls (Additional file 2). IPA was used to predict activation and/or inhibition of canonical pathways and processes and it predicted the activation of the sirtuin signalling pathway (Z-score = 1, *p*-value = 4.0 × 10^–^6; Fig. 3C). Experimental values of proteins associated with the sirtuin pathway (e.g. ACLY, GLS, MAPK1, MT-ND5, NAMPT, NDRG1, PFKM, PGAM1, PGK1, TUBA3E and VDAC3) were obtained from the quantitative analysis (Fig. 3D). Based upon the Ingenuity curated database, other biological networks and functions predicted to be affected by valproate treatment included the inhibition of the EIF2 signaling pathway (Z-score = − 1.89, *p*-value = 2.21 × 10^–6^, Fig. 3C, Additional file 3) and inhibition of “degeneration of neurons” (Z-score = − 1.062, *p*-value = 2.2 × 10^–4^), “apoptosis” (Z-score = − 1.696, *p*-value = 3 × 10^–5^) (Additional file 4).

### Valproate treatment induces increased activity of the sirtuin pathway, which has protective effects for MJD zebrafish motor function

**Fig. 4:**
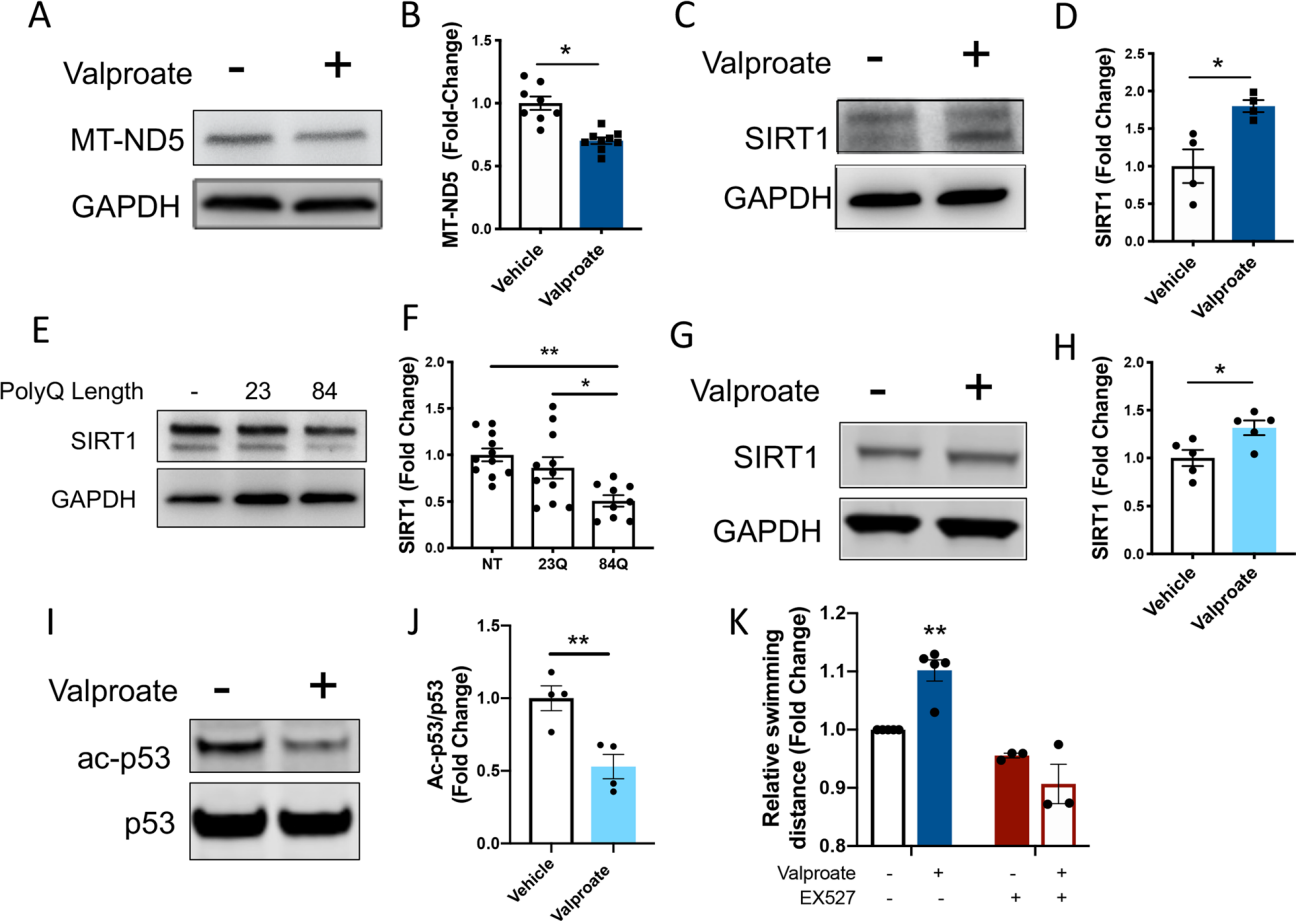
Sodium valproate treatment increases SIRT1 levels and signs of sirtuin activity, validating the increased sirtuin activity predicted by mass spectrometry. **A** Valproate treatment predicted decreased MTND5 levels from the proteomic analysis. Immunoblot of MTND5 showed valproate treated EGFP-ataxin-3 84Q protein lysates at 6dpf were decreased. **B** Quantification of MTND5 levels confirmed this finding (*p < 0.001, n = 8–9). **C** Immunoblots of mutant ataxin-3 zebrafish treated with valproate showed an increase in SIRT1 expression. **D** Quantification of SIRT1 levels revealed an increase with valproate treatment (*p = 0.015, n = 4). **E** Immunoblots of 6-day old transgenic MJD zebrafish show levels of SIRT1. **F** Densitometric analysis revealed decreased levels of SIRT1 in zebrafish expressing ataxin-3 with polyQ expansion compared to wild-type ataxin-3 and non-transgenic fish (*p* = 0.025 and *p* = 0.002 respectively, n = 9–11). **G** Immunoblot analysis of ataxin-3 84Q expressing HEK293 cells treated with valproate revealed that valproate increases SIRT1. **H** Quantification SIRT1 levels revealed a significant increase in SIRT1 from valproate treatment (*p* = 0.024, n = 5). **I** Immunoblot of acetylated p53 and p53, as p53 deacetylation is a marker of sirtuin activity from ataxin-3 84Q expressing HEK293 cells treated with and without valproate. **J** Quantification of acetylated p53 levels, normalized to p53 levels, revealed that valproate treatment resulted in increased p53 deacetylation (*p* = 0.008, n = 4). **K** Whilst treating the EGFP-ataxin-3 84Q zebrafish with valproate resulted in the zebrafish swimming longer distances, co-treatment with valproate and EX527, or EX527 alone (SIRT1 inhibitor), did not result in increased swimming distances (*p* < 0.002; valproate group significantly greater distances swum than all other groups). Data represents mean ± SEM. All cell culture experiments are of independent experiments. Comparisons between vehicle and valproate treatment were analysed statistically using unpaired student t-tests, comparison between ATXN3 genotypes were analysed using an unpaired one-way ANOVA followed by a Tukey post-hoc analysis and comparison of valproate versus EX527 treated was analysed using a two-way ANOVA followed by Tukey post-hoc analysis

Label free quantitative (LFQ) and IPA analysis of canonical signaling pathways of valproate treatment on EGFP-Ataxin-3 84Q zebrafish predicted an upregulation of the sirtuin pathway. We firstly validated the proteomic findings of increased predicted activation of the sirtuin pathway, including a predicted decrease in the level of mitochondrially encoded NADH Subunit 5 (MT-ND5, or ubiquinone) protein in valproate treated MJD zebrafish, and found this to be true to the prediction (Fig. 4A, B). Additionally, we found that SIRT1 levels were indeed increased by 1.7-fold in lysates from valproate treated EGFP-Ataxin-3 84Q zebrafish compared to the vehicle treated controls (Fig. 4C, D). Interestingly, comparison of the levels of SIRT1 present in the different transgenic ataxin-3 zebrafish genotypes (prior to valproate treatment) at 6 dpf revealed a decrease in SIRT1 levels in zebrafish expressing polyQ expanded ataxin-3 compared to the those expressing wild-type human ataxin-3 and non-transgenic zebrafish (Fig. 4E, F).

In a similar way, we found that expression of human ataxin-3 84Q in HEK293 cells resulted in decreased SIRT1 levels compared to in cells expressing human ataxin-3 28Q (Additional file 5). We also demonstrated an increase in SIRT1 band intensity in HEK293 cells expressing mutant ataxin-3 (84Q) treated with valproate compared to those receiving vehicle treatment (Fig. 4G, H). As SIRT1 is a deacetylase, we also examined the level of acetylation of p53, a known substrate of SIRT1 deacetylation. Immunoblotting revealed a decrease in acetylated p53 levels upon valproate treatment compared to the vehicle control (Fig. 4I, J).

Finally, we co-treated the mutant ataxin-3 zebrafish with both valproate and the SIRT1 inhibitor, EX527 (12.5 µM), to determine whether the beneficial effect of valproate on zebrafish swimming were indeed through activity of the sirtuin pathway. Treating the MJD zebrafish with valproate resulted in an increase in the distance swum by the zebrafish, compared to vehicle treated MJD zebrafish. Co-treatment with valproate and EX527 prevented that increase in swimming distance, suggesting that the beneficial effect of valproate on the MJD zebrafish was dependent on activity of the sirtuin pathway (Fig. 4K).

### Confirmation of protective effect of activating sirtuin pathway in MJD zebrafish through resveratrol treatment

**Fig. 5:**
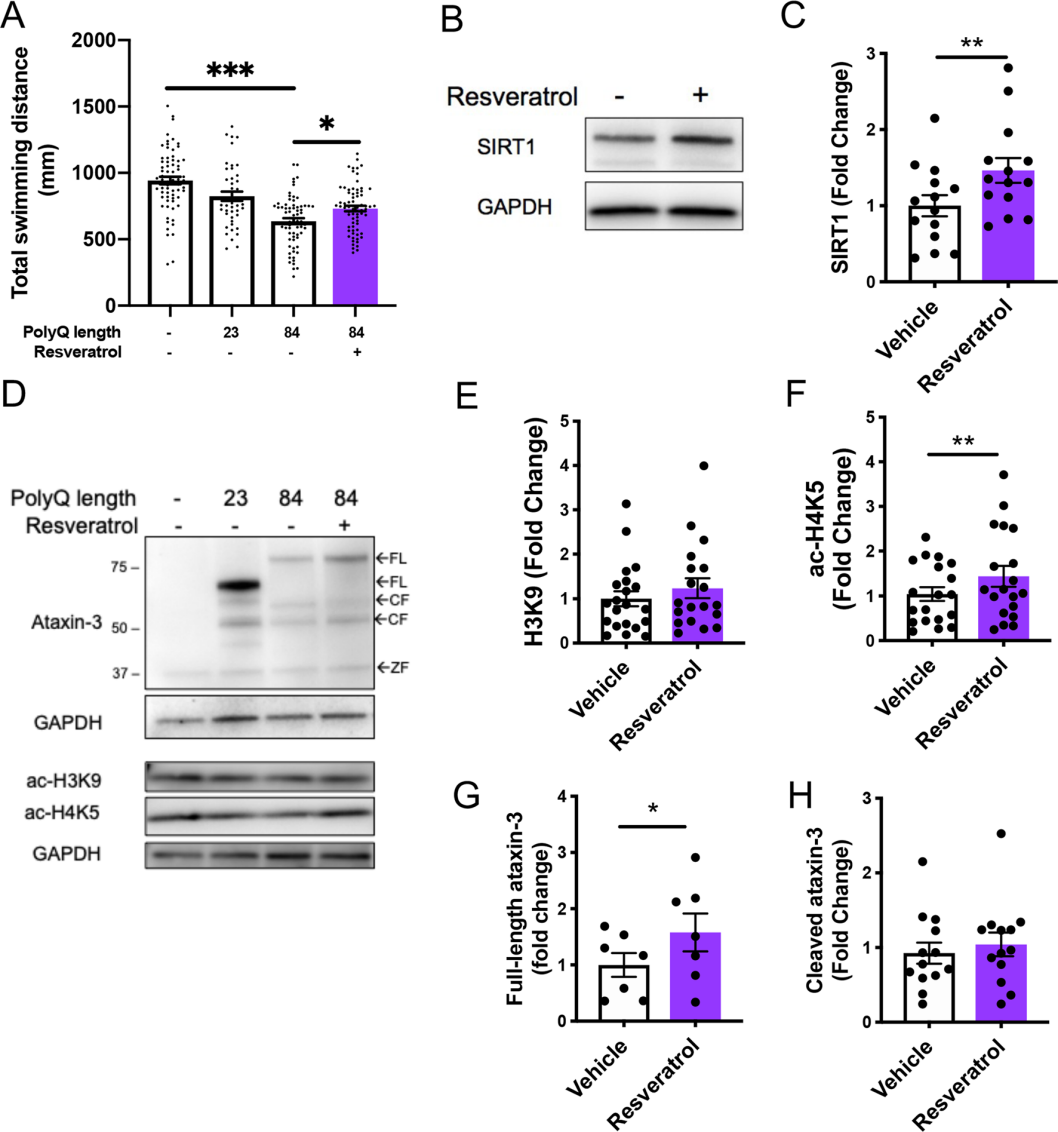
Resveratrol treatment alleviates motor dysfunction whilst simultaneously increasing acetylated histone and SIRT1 levels. **A** Mutant ataxin-3 zebrafish at 6 days post fertilization (dpf) showed a decrease in the distance swum (***p < 0.001) whilst resveratrol treatment (50 µM) from 1–6 dpf rescued this dysfunction, (*p = 0.0033, n = 45–71). **B** Immunoblot of vehicle versus resveratrol treated EGFP-Ataxin-3 84Q fish at 6 dpf showed increased SIRT1 levels following resveratrol treatment. **C** Quantification revealed that SIRT1 levels were increased by resveratrol (*p* = 0.003, n = 14). **D** Immunoblot of 6 dpf transgenic MJD zebrafish treated with or without resveratrol increases full-length (FL) human ataxin-3 levels. **E** Quantification of levels of acetylated H3K9 (ac-H3K9); **F** acetylated H4K5 (ac-H4K5); **G** FL ataxin-3; and **H** cleaved (CF) ataxin-3, revealed that resveratrol treatment produced no change in ac-H3K9 levels, an increase in ac-H4K5 (**p = 0.005), an increase in FL-ataxin-3 (*p = 0.018) and no change in cleaved ataxin-3 levels (n = 19, n = 19, n = 7 and n = 7 respectively). *CF* cleavage fragment, *ZF* zebrafish. Data represents mean ± SEM. Statistical analysis used for motor behaviour tracking was a one-way ANOVA followed by a Tukey post-hoc analysis and the immunoblot comparisons were analysed using a paired student *t*-test

To confirm that increased activity of the sirtuin pathway can improve movement of the transgenic MJD zebrafish, we treated our mutant ataxin-3 zebrafish with resveratrol, a natural product known to also activate the sirtuin pathway [11, 19]. Treating the mutant ataxin-3 zebrafish with resveratrol (50 µM) for five days significantly increased the distances swum by the zebrafish larvae compared to those treated with vehicle control (Fig. 5A). Interestingly, treating non-transgenic zebrafish with resveratrol also increased the distance swum by those zebrafish compared to that swum by vehicle treated controls (Additional file 6). Resveratrol treatment also resulted in an increase in levels of SIRT1 protein in the EGFP-Ataxin-3 84Q larvae compared to those receiving vehicle control (Fig. 5B, C). Interestingly, whilst immunoblots of resveratrol treated zebrafish did not show differences between acetylated histone 3 (Fig. 5D, E), there was an indication of increased levels of acetylated histone 4 (Fig. 5F) and full-length human ataxin-3 (Fig. 5G). As with valproate treatment, resveratrol treatment did not affect the levels of cleaved human ataxin-3 (Fig. 5H).

### Sodium valproate treatment induces macroautophagy in a sirtuin dependent manner

**Fig. 6:**
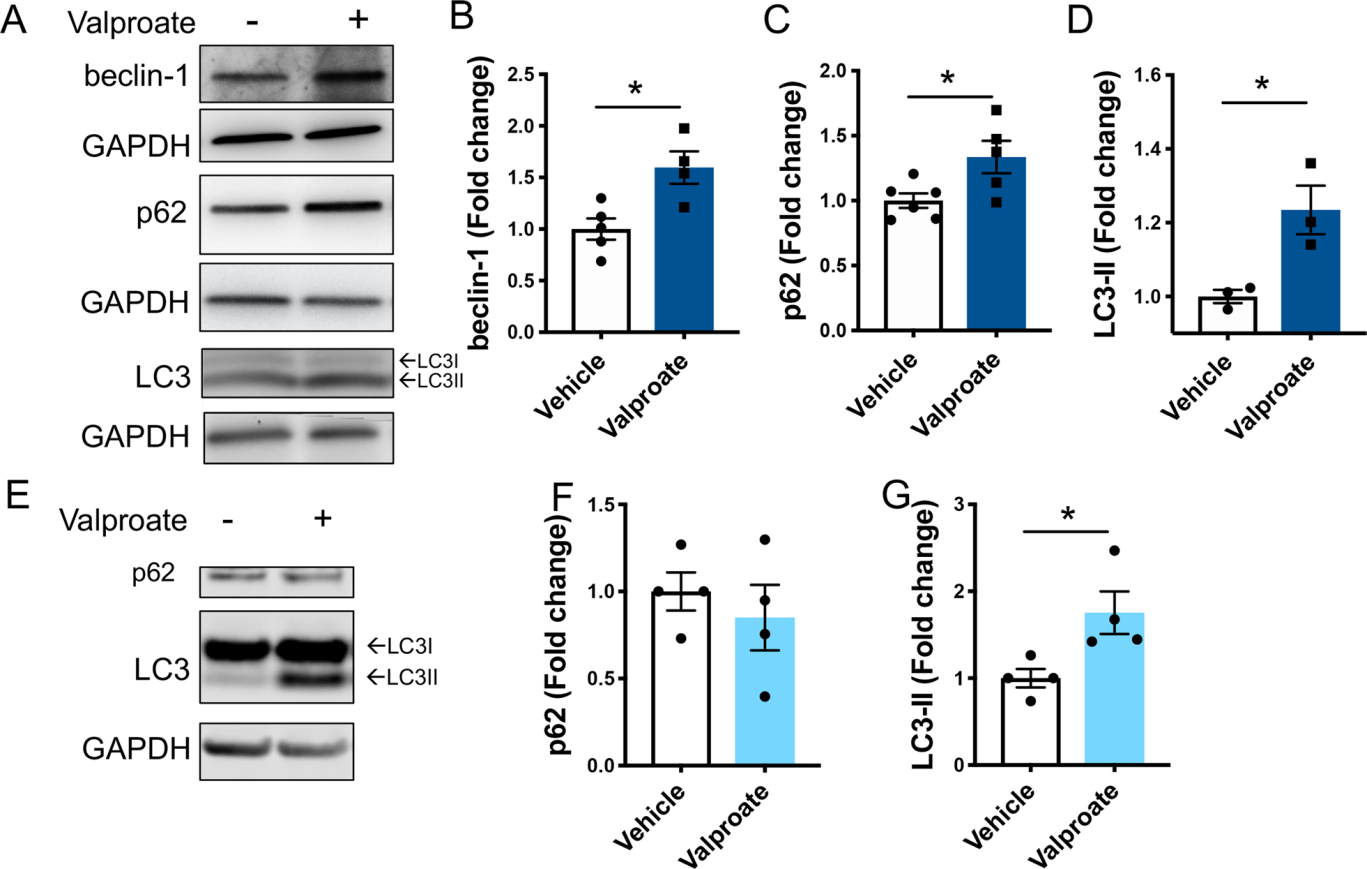
Treatment with sodium valproate (valproate) induces activity of the autophagy pathway in transgenic MJD zebrafish and human ataxin-3 expressing HEK293 cells. **A** Immunoblots of 6 dpf EGFP-Ataxin-3 84Q zebrafish treated with either valproate or vehicle control were probed with several markers of the autophagy pathway. Quantification of **B** beclin-1, **C** p62 and **D** LC3-II each revealed a significant increase with valproate treatment (*p = 0.014, *p* = 0.029 and *p* = 0.026 respectively, n = 3–6). E) Valproate treatment of HEK293 cells expressing Ataxin-3 84Q resulted in similar p62 levels, but increased LC3II levels. Quantification of these substrates revealed **F** p62 was not significantly different between valproate and vehicle treatment (*p* = 0.518) whilst **G** LC3II levels were significantly different for valproate compared to vehicle treated (*p* = 0.031, n = 4). Comparisons between vehicle and valproate treatment were compared using unpaired student *t*-tests

Sodium valproate is a known inducer of activity of the macroautophagy (autophagy) protein quality control pathway [49]. Increased activity of the sirtuin pathway has also been demonstrated to induce increases in autophagy [20, 21]. We therefore examined whether markers of autophagy activity (beclin-1, p62 and LC3II) were elevated following the valproate treatment of the MJD zebrafish larvae and HEK293 cells. Valproate treatment of the MJD zebrafish resulted in increased levels of beclin-1, p62 and LC3II compared to the vehicle-treated control (Fig. 6A–D), supporting increased autophagy induction. HEK293 cells stably expressing ataxin-3 84Q treated with valproate had similar levels of p62 to vehicle treated cells (Fig. 6E, F) but did show an increase in LC3II levels (Fig. 6E, G).

**Fig. 7:**
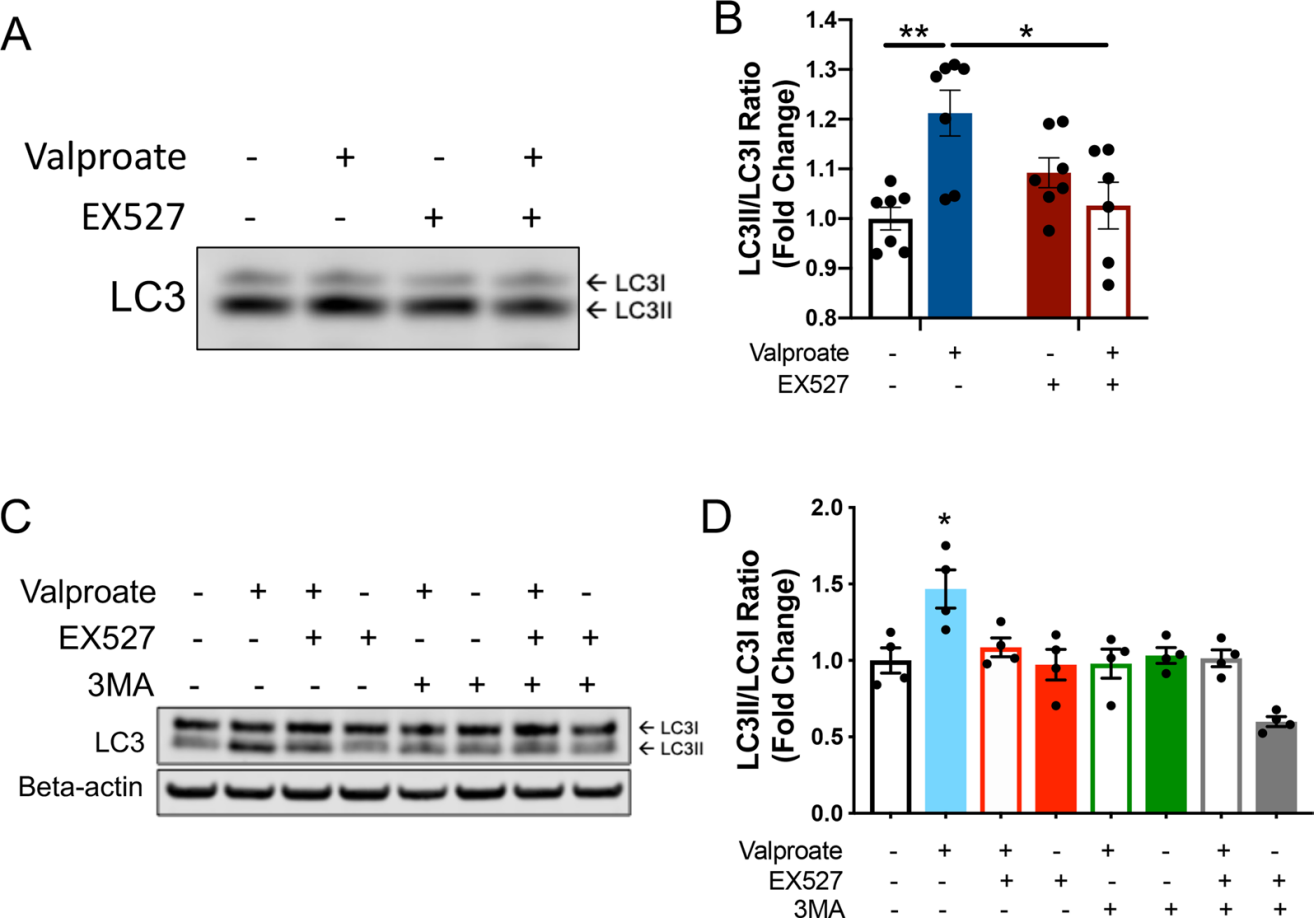
Induction of autophagy following sodium valproate (valproate) treatment of MJD zebrafish and human ataxin-3 expressing HEK293 cells is dependent on sirtuin activity. **A** Protein lysates from groups of MJD zebrafish larvae underwent immunblotting for LC3B. **B** Densiometric analysis revealed that valproate treatment increased LC3II/I ratio compared to vehicle treatment (**p = 0.003), but co-treatment with valproate and EX527 prevented this increase in LC3II/I (*p = 0.013, n = 6–7). **C** Immunoblots for LC3 were performed on lysates from cells treated with valproate, EX527 and 3MA, together and alone. **D** Densiometric analysis revealed that whilst valproate increased LC3II/I ratio, suggesting increased autophagosome production, cotreatment with valproate and EX527 or valproate and 3MA prevented that increase (*p < 0.05, n = 3 independent experiments). Data represents mean SEM. Comparisons were made using two-way ANOVAs followed by a Tukey post-hoc analysis

We found that co-treating the MJD zebrafish with valproate and EX527 prevented the increased LC3II/I ratio produced by valproate treatment alone (Fig. 7A, B), suggesting that the increased formation of autophagosomes produced by valproate was dependent on sirtuin activity. Likewise, co-treating the HEK293 cells expressing ataxin-3 84Q with valproate and EX527 (20 µM) or valproate (3 mM) and the autophagy inhibitor 3MA (5 mM), prevented the increased LC3II/I ratio resulting from valproate treatment alone (Fig. 7C, D), supporting that the induction of autophagy by valproate treatment is dependent on SIRT1 activity.

**Fig. 8:**
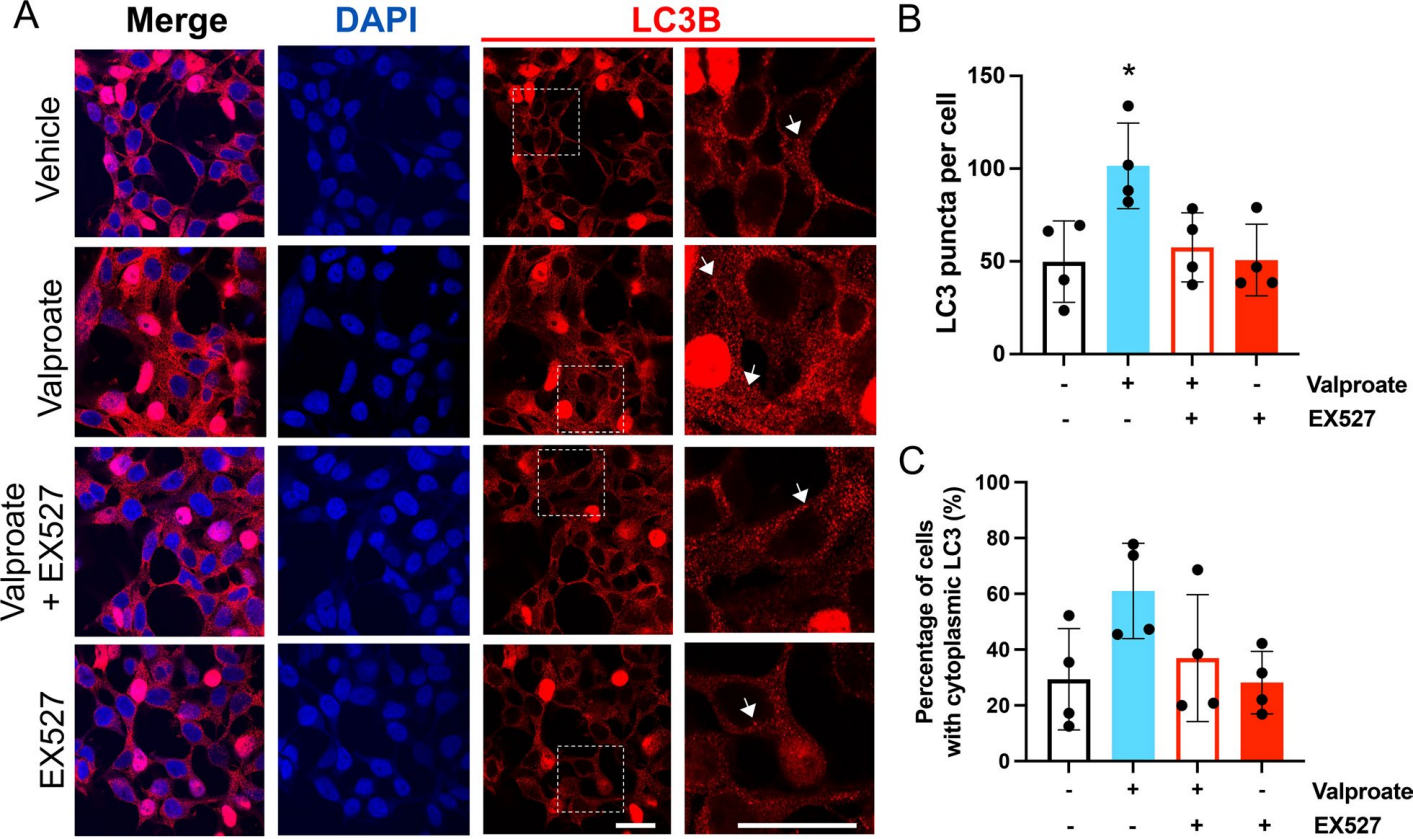
Treatment with sodium valproate (valproate) results in an increase in LC3 within HEK 293 cells expressing ataxin-3 84Q. **A** HEK 293 cells expressing human ataxin-3 84Q were treated with vehicle, sodium valproate (valproate), valproate and EX527 or EX527 alone, and afterwards stained for LC3 (red) and nuclei (DAPI, blue). Those treated with valproate showed strong LC3 (red) staining. Enlarged inset images of the LC3 staining make the LC3 puncta (examples marked with arrows) easier to see. **B** Automated counting of LC3 (red) puncta revealed that valproate treatment increased the presence of LC3 puncta, whilst EX527 cotreatment and EX527 alone had similar numbers of puncta to the vehicle control treated group (*p < 0.0483, valproate compared to all other groups). **C** Manual counting of cells with cytoplasmic LC3 (red) staining revealed that valproate had not produced a significant cytoplasmic shift of LC3. Comparisons were analysed using a one-way ANOVA followed by a Tukey post-hoc analysis. Scale bars represent 20 μm

To visualize these changes in LC3 levels we performed immunofluorescent staining of HEK293 cells stably expressing Ataxin-3 84Q treated with valproate (3 mM) and/or EX527 (20 µM). It has previously been demonstrated that SIRT1 increases autophagy activity by deacetylating autophagic proteins such as LC3 and increasing relocation of LC3 from the nucleus to cytoplasm [20]. Our staining revealed that valproate treatment produced a robust increase in the number of LC3 puncta present in the cells, and that EX527 co-treatment prevented that increase (Fig. 8A, B). Whilst the immunostaining images suggested that valproate treatment had caused the LC3 to shift to the cytoplasm, manual counting of cells with cytoplasmic LC3 did not detect a statistically significant increase with valproate treatment (Fig. 8C). Collectively these findings confirm that valproate treatment does increase the presence autophagosomes in a SIRT1-dependent manner.

## Discussion

Here we demonstrate that histone acetylation, which plays a role in transcription regulation, is altered in transgenic MJD zebrafish expressing human ataxin-3 with expanded polyQ tract. Zebrafish expressing EGFP-Ataxin-3 84Q appeared to have decreased acetylated histones 3 and 4 compared to non-transgenic zebrafish, suggesting that presence of polyQ expansion ataxin-3 may affect the regulation of histone acetylation by ataxin-3 protein. This finding is in line with previous studies by Yi et al., [50] and Chou et al., [8]. However, it is in contrast with Esteves et al. [13] and Evert et al., [14], who found similar or higher levels of histone acetylation, respectively, in cells expressing polyQ expanded ataxin-3. As the levels of acetylated histones 3 and 4 were compared to a loading control and not to the total histone protein, the level of histone acetylation present in transgenic MJD zebrafish cannot be concluded.

We used this model, which has benefits of rapid drug testing, to examine whether treatment with sodium valproate, a class I and IIa HDAC inhibitor, would be beneficial for this MJD model. Valproate has previously been explored as a potential therapeutic agent for MJD [22], along with other neurodegenerative diseases such as Huntington’s disease [51], amyotrophic lateral sclerosis [4], Parkinson’s disease [18] and Alzheimer’s disease [34]. Treatment with valproate, amongst other HDAC inhibitor compounds, has been demonstrated to positively modify disease phenotypes in other models of MJD including cellular models, *Drosophila* and mice [8, 24, 46, 50]. In contrast, Esteves et al. [13] have previously reported that valproic acid had limited benefit for a mouse model of MJD. One trial has reported that treating MJD patients with valproate is safe and efficacious [22]. Testing of valproate treatment for another polyQ repeat disease, Huntington’s disease, reported a dose dependent alleviation of hyperkinesia, which is hypothesized to be due to an action on GABA transmission [37].

In our study, treatment with a low dosage of valproate led to improve swimming behavior in zebrafish overexpressing mutant ataxin-3. Valproate treatment also produced an increase in levels of acetylated histone 3 and high-dose valproate increased levels of acetylated histone 4. These results of increased acetylated histones align with previous findings of a beneficial effect of increased histone acetylation following HDAC inhibitor treatment for models of MJD [24, 46, 50]. Importantly, we also found that high concentrations of valproate were not protective in our model, and in fact resulted in morphological abnormalities, decreased movement and decreased survival of the zebrafish. This may suggest that there is a dosage threshold for valproate treatment and that dose optimisation is an important consideration for future investigations. The dose found to be effective within this study (3.125 μM) can be calculated to be the equivalent of a 200 mg dose of sodium valproate in a human patient, however the ease of absorption of drugs and absence of a developed blood brain barrier in zebrafish larvae at the age of our testing, suggest that our dose would be the equivalent of higher dose in human patients.

Unexpectedly, treatment with valproate produced increased expression of human ataxin-3 in the EGFP-ataxin-3 84Q zebrafish and HEK293 cells, perhaps due to an effect of valproate on transcription regulation. The ability of valproate to provide neuroprotection despite this increase in pathogenic ataxin-3 expression prompted us to explore whether valproate additionally activates neuroprotective pathways that alleviate the stresses induced by the expression of mutant ataxin-3. Accordingly, using proteomic protein profiling techniques and pathway analysis, we identified that the valproate treatment had produced activation of the sirtuin pathway. This finding was confirmed through immunoblot analysis for SIRT1 in lysates from valproate treated zebrafish and HEK293 cells expressing ataxin-3 84Q. Another protein predicted to be downregulated by valproate that was validated in the MJD zebrafish was MTND5. Recently, it has been shown that MTND5 was downregulated in plasma blood mononuclear cells of a patient with haemorrhagic stroke after a single treatment of valproic acid (140 mg/kg) [3]. We also found that valproate treatment of HEK293 cells resulted in decreased levels of acetylated p53, a marker of increased sirtuin activity. We further examined whether SIRT1 protein levels were affected by expression of the polyQ expanded ataxin-3 in the stably expressing HEK293 cells and transgenic MJD zebrafish. We found decreased levels of SIRT1 in HEK293 cells expressing polyQ expanded ataxin-3 compared to wild-type ataxin-3 and zebrafish expressing polyQ expanded human ataxin-3 compared to those expressing wild-type human ataxin-3 or non-transgenic controls. This finding is in line with Cunha-Santos et al. [11], wherein mice expressing the mutant form of ataxin-3 contain decreased levels of SIRT1 protein in the cerebellum. Together these findings indicate that treatment with valproate can rectify a decrease in SIRT1 levels present in MJD zebrafish.

To test whether this increased sirtuin activity was important for the improvement in the swimming of the MJD zebrafish resulting from valproate treatment we performed a co-treatment study involving valproate along with the SIRT1 inhibitor, EX527. We found that co-treatment with EX527 prevented the improvement in swimming seen with valproate treatment alone, supporting that the protective effect was SIRT1-dependent. Further, we found that treatment with resveratrol, a natural compound known to increase longevity through upregulation of SIRT1 levels, also improved the swimming of the MJD zebrafish. Together these findings indicate that induction of the sirtuin pathway is beneficial for the swimming of the MJD zebrafish.

Our findings of a beneficial effect of valproate and resveratrol treatment in our zebrafish model of MJD, along with increasing SIRT1 activity for each, is consistent with Cunha-Santos et al. [11] previous findings that elevating SIRT1 levels in transgenic MJD mice via gene delivery or resveratrol treatment results in decreased neuropathology and motor dysfunction, respectively [11]. A beneficial effect of elevating SIRT1 levels is also in line with previous reports that SIRT1 expression is decreased in MJD models [11, 48], which we also confirmed in our MJD zebrafish and stable HEK293 cell models. This decreased level of SIRT1 expression may be explained by previous findings that polyQ-expanded ataxin-3 and huntingtin proteins have been shown to interact with cAMP response element binding (CREB) protein [42], a transcription factor known to regulate levels of SIRT1 [17, 30]. Interestingly, valproate treatment has been shown to activate the CREB pathway [24, 40], which may explain the increase in SIRT1 levels produced by valproate treatment here. Nevertheless, further investigation into the mechanisms by which valproate increases activity of the sirtuin pathway is required.

To further explore the mechanisms of the protective effect of valproate treatment on the MJD zebrafish, we examined whether markers of autophagy were elevated in the valproate treated MJD zebrafish and HEK293 cells. Valproate treatment has previously been demonstrated to induce activity of the autophagy pathway to produce neuroprotective effects in neurodegenerative disease models [44, 52]. Likewise, increased levels of SIRT1 have been reported to increase autophagic activity, through the removal of the acetyl groups from autophagic substrates Atg5, Atg7 and LC3 [20, 21, 25]. Furthermore, a recent study of patient serum following a single dose of valproic acid (140 mg/kg) reported an upregulation in the autophagy pathway [3]. The present study provides evidence of autophagy induction following valproate treatment in both the mutant ataxin-3 zebrafish and treated HEK293 cells expressing polyQ expanded ataxin-3. Further, we found that the increased autophagic activity produced by valproate treatment in the HEK293 cells and MJD zebrafish were both dependent on SIRT1 activity, as co-treatment of valproate and the SIRT1 inhibitor EX527 prevented the induction of autophagy. Analysis of LC3B immunoreactivity in HEK293 cells expressing Ataxin-3 84Q revealed that valproate treatment had produced a significant increase in LC3. However, this effect was abolished when valproate treatment was combined with EX527, suggesting the increase in LC3 produced by valproate treatment was mediated by the sirtuin pathway. Huang et al. [20] had previously reported that SIRT1 overexpression, resveratrol treatment and starvation each cause SIRT1 to deacetylate LC3, driving nuclear export of LC3 to aid formation of autophagosomes. In our study we failed to detect a robust shift of LC3 from the nucleus to the cytoplasm, however this may have been due to imaging limitations.

In our study, we see a simultaneous increase in levels of full-length human ataxin-3 protein and induction of autophagy following treatment with valproate. We hypothesise that the increased full-length human ataxin-3 may occur due to the transcriptional effects of valproate treatment. Interestingly, Yi et al. [50] have previously reported that valproate treatment has protective effects on a Drosophila model of MJD, decreasing apoptosis and alleviating climbing disability, without decreasing the presence of ataxin-3 protein aggregates. Nevertheless, future investigation into the effect of valproate treatment on presence of ataxin-3 protein aggregates would be valuable.

## Conclusion

Our findings demonstrate for the first time that sodium valproate treatment acts to increase activity of the neuroprotective sirtuin signaling pathway. We identified that the ability of valproate to induce increased activity of the autophagy pathway is dependent on activity of the sirtuin pathway. Further, our studies identified that valproate, along with the sirtuin inducer resveratrol, both improved the movement of our MJD zebrafish. We found that this beneficial effect of valproate treatment on the transgenic MJD zebrafish was indeed occurring through activity of the sirtuin pathway. These findings are promising because both sodium valproate and resveratrol have potential to be safe for human treatment as sodium valproate already has FDA approval to treat other neurological conditions and resveratrol is a natural phenol found in the skin of grapes and berries. Further testing of sodium valproate, resveratrol, and other candidates that target the sirtuin pathway, would be beneficial for the development of treatments for MJD, and other related neurodegenerative diseases.

## Methods

### Transgenic zebrafish lines

All animal experiments were performed in accordance with the Animal Ethics Committee of the University of Sydney (K00/2014/575 & 576) and the Animal Ethics Committee of Macquarie University, N.S.W., Australia (ARA: 2016/04 and 2017/019). Zebrafish were housed in a standard recirculating aquarium system at 28.5 °C with a 12-h light and 12- hour dark cycle. Transgenic MJD zebrafish lines were generated as described previously [47], involving crossing of a driver line: Tg(*elavl3*:Gal4-VP16; mCherry) with responder lines Tg(UAS:dsRed,EGFP-ATXN3_Q23) or Tg(UAS:dsRed,EGFP-ATXN3_Q84) to achieve expression of EGFP-fused human ataxin-3 containing either 23Q or 84Q. These F1 fish were then in-crossed and their offspring (male and female) were used for the drug treatment studies at the larval stage.

### Motor function testing

All zebrafish behavioral tracking was performed using a ZebraLab Tracking System (Viewpoint) including a ZebraBox for tracking larvae. Tracking of 6-day post fertilization (dpf) larvae was conducted in 24 multi-well plates within a ZebraBox housed with an enhanced light source, under conditions of 6 min light, 4 min dark and 4 min light. The total distance travelled by each larva within the dark phase was calculated.

### Drug treatment studies

Zebrafish embryos (24 hpf) were screened for fluorescence (EGFP and dsRED) indicating that they were positive for the transgenes. Embryos positive for EGFP-Ataxin-3 84Q, of similar expression, were divided into equal numbers and treated for five days with sodium valproate (3.125 µM or 6.25 µM, solubilized in E3 medium), EX527 (12.5 µM, solubilized in DMSO) or resveratrol (50 µM, solubilized in ethanol) through addition of the drug to the E3 buffer that the larvae were incubated in [31]. Control treated animals (all genotypes: EGFP-Ataxin-3 23Q, 84Q and non-transgenic control) received the equivalent volume of appropriate vehicle (E3, DMSO or ethanol, respectively). Behavioural testing was performed on morphologically normal larvae at 6 dpf within the ZebraBox.

### Western blotting

Protein lysates were prepared following euthanasia of zebrafish larvae (6 dpf) in RIPA buffer containing protease inhibitors (Complete ULTRA Tablets, Roche), followed by homogenisation via probe sonication. Equal amounts of protein were separated via SDS-PAGE and transferred to PVDF membrane for immunoblot probing. For cell culture lysates, cells were lysed at 3 days in ice-cold RIPA buffer containing protease inhibitors (Roche) and protein concentration was determined by BCA Assay (Pierce^TM^, ThermoFisher).

Antibodies used included rabbit anti-MJD (kind gift from H. Paulson), rabbit anti-acetylated histone 3 and 4 (acetylated H3K9 and H4K5 antibodies, Cell Signaling), mouse anti-histone 4 (Cell Signaling), rabbit anti-MT-ND5 (Abcam), rabbit anti-SIRT1 (Aviva Systems Biology), rabbit anti-beclin-1 (Proteintech), rabbit anti-p62 (MBL Life Science), mouse anti-p62 (Abcam), rabbit anti-LC3B (Abcam), rabbit anti-acetylated p53 (K382; Abcam), rabbit anti-p53 (Abcam), mouse anti-beta-actin (Sigma) and mouse anti-GAPDH (Proteintech). The immunoblots were probed with appropriate secondary antibodies (Promega) and visualised by chemiluminescence (Supersignal detection kit, Pierce) using a BioRad GelDoc System or via fluorescence using the LiCor Odyssey System. The intensities of the bands were quantified by Image Studio Lite and the target protein expression level was determined by normalizing against the loading control protein.

### In-gel trypsin digestion

Whole lysates of 6 dpf EGFP-Ataxin-3 84Q (n = 3 for vehicle and valproate treatment) were separated by SDS-PAGE for 5 min (~ 1 cm into the gel) and stained with Coomassie Brilliant Blue. Stained proteins were excised from the gel and prepared for in-gel trypsin digestion as described [39]. Briefly, gel pieces were equilibrated and dehydrated with 50 mM NH_4_HCO_3_ pH 7.8 and 50 mM NH_4_HCO_3/_50% (v/v) acetonitrile pH 7.8 respectively and dried under vacuum centrifugation. The protein gel pieces were reduced with 10 mM DTT at 55 °C for 30 min and alkylated with 20 mM iodoacetamide (IAA) for 1 h in the dark at room temperature. The proteins were digested with trypsin (12.5 ng/µl) overnight at 37 °C. Following digestion, tryptic peptides were passively diffused in the presence of 50% (v/v) acetonitrile/2% (v/v) formic acid (2×) in a bath sonicator and collected. The acetonitrile was evaporated by vacuum centrifugation, and tryptic peptides were desalted on a pre-equilibrated C_18_ Sep-Pak cartridge and eluted in 50% (v/v) ACN, 0.1% (v/v) formic acid, and dried under vacuum centrifugation.

### Reverse phase (C_18_) liquid chromatography mass spectrometry (LC–MS/MS)

Peptide fractions were separated on a nanoLC system (Thermo) employing a 100-min gradient (2–50% v/v acetonitrile, 0.1% v/v formic acid for 95 min followed by 90% v/v acetonitrile, 0.1% v/v formic acid for 5 min) with a flow rate of 300 nL/min. The peptides were eluted and ionized into Q-Exactive mass spectrometer (Thermo). The electrospray source was fitted with an emitter tip 10 μm (New Objective, Woburn, MA) and maintained at 1.8 kV electrospray voltage. FT-MS analysis on the Q-Exactive was carried out with a 70,000 resolution and an AGC target of 1 × 10^6^ ions in full MS (scan range m/z 350–1800); and MS/MS scans were carried out at 17,500 resolution with an AGC target of 2 × 10^5^ ions. Maximum injection times were set to 60 and 100 ms respectively. The ion selection threshold for triggering MS/MS fragmentation was set to 15,000 counts and an isolation width of 2.0 Da was used to perform HCD fragmentation with normalised collision energy of 30%.

Spectra files were processed using the Proteome Discoverer 1.4 software (Thermo) incorporating the Mascot search algorithm (Matrix Sciences, UK) and the *Danio rerio* NCBI RefSeq protein database (05/05/2017, Sequences 46,751). Peptide identifications were determined using a 20-ppm precursor ion tolerance and a 0.1-Da MS/MS fragment ion tolerance for FT-MS and HCD fragmentation. Carbamidomethylation modification of cysteines was considered a static modification while oxidation of methionine and acetyl modification on N-terminal residues were set as variable modifications allowing for maximum three missed cleavages. The data was processed through Percolator for estimation of false discovery rates. Protein identifications were validated employing a q-value of 0.01 (1% false discovery rate).

Label-free quantitative proteomics were carried out by calculating the normalised spectral abundance factors (NSAF) according to Zybailov et al (2006) [53], which takes into account the length of a given protein as well as the total amount of protein in a given sample. A fraction (0.5) of a spectral count was added to all samples to account for missing values and total spectral counts for at least one condition were set to a minimum of 5. NSAF values were log_2_-transformed and Student’s t-tests were used to identify significant (P ≤ 0.05) changes in protein abundance between transgenic zebrafish expressing EGFP-Ataxin-3 84Q treated with vehicle and valproate. Statistical data preparation and tests were done using Microsoft Excel. Enriched GO annotations and signaling pathways were identified using the Database for Annotation, Visualization and Integrated Discovery (DAVID) and Ingenuity® Pathway Analysis (IPA; QIAGEN) to predict activation and inhibition of cellular pathways upon treatment. The filtering criteria for IPA core analysis used experimental fold change cut offs of -1.5 fold (downregulated) and 1.5-fold (upregulated) with p-value < 0.05, which was analysed using IPA content version 436,056,602 (Release date: 28^th^ March 2018).

The mass spectrometry proteomics data has been deposited to the ProteomeXchange Consortium via the PRIDE [43] partner repository with the dataset identifier PXD009612 (Username: reviewer27566@ebi.ac.uk; Password: ta6siSAA).

### Sodium valproate treatment of HEK293 cells expressing human ataxin-3

Wildtype HEK293 cells were grown in DMEM supplemented with 10% fetal bovine serum and maintained at 37 °C and 5% CO_2_. Wild type HEK293 cells were transfected with Lipofectamine LTX with plus reagent (Thermoscientific) and 5 µg of DNA from pcDNA3.1 vectors containing full length human ataxin-3 (containing a polyQ stretch of 28 glutamines or 84 polyglutamines) and a neomycin resistance gene. Cells were treated with 500 μg/mL of Geneticin (Sigma Aldrich), a selective antibiotic analogue of neomycin, to select cells stably expressing pcDNA3.1 vectors. Stable transgenic expression of ataxin-3 or an empty vector control was maintained via treatment with 250 µg/mL Geneticin.

For valproate treatments, cells were seeded into 24 well plates at a density of 30,000 cells/cm^2^ and left overnight in a 37 °C incubator supplemented with 5% CO_2_. After 24 h, cells were treated with valproate (3 mM), EX527 (20 μM) or vehicle control in complete growth media. Growth media containing the drug compound was replenished daily for 3 days.

### Immunostaining and analysis of subcellular localization of LC3 in HEK 293 cells expressing human ataxin-3

For immunostaining, coverslips (n = 3 per condition) were fixed with 4% paraformaldehyde (20 min) and permeabilised with 0.2% Triton X-100 in PBS. Coverslips were briefly washed (2 × 5 min in PBS) and incubated in 2% BSA in PBS to block against non-specific binding and incubated in 2% BSA containing rabbit anti-LC3B primary antibody (Abcam). Coverslips were then washed 3 × 5 min in PBS, followed by incubation with goat anti-rabbit Alexa 555 secondary antibody (Life Technologies). Coverslips were washed 3 × 5 min in PBS and finally mounted onto glass slides using Prolong Gold mounting medium containing 4′,6-diamidino-2-phenylindole (DAPI) (Thermofisher). Images were acquired via confocal microscopy using a Zeiss LSM-880 confocal microscope (Plan-Apochromat 40x/1.3 Oil DIC UV-IR M27objective, master gain: 800) running Zen Black software (Zeiss, Gottingen, Germany). To visualize LC3 staining, a DPSS561 laser was used (5.6% laser power) and a UV laser (3% laser power) was used the visualize DAPI-positive nuclei. Images were processed using Airyscan mode and brightness and contrasted was adjusted in an identical manner.

The number of LC3 stained puncta was quantified in an automated manner within ImageJ, through use of the particle analysis function to count LC3 stained (red) particles, with the number of puncta per image divided by the number of DAPI stained (blue) nuclei per image. Manual counting of the number of cells with cytoplasmic LC3-staining, per image, was performed by an experimenter blind to experimental group. The percentage of cells with cytoplasmic LC3-staining was calculated by dividing those counts by the total number of DAPI stained nuclei per image.

### Statistics

Data analysis was performed using GraphPad Prism (version 8). Densitometric analysis of vehicle treatment versus valproate (or resveratrol) were compared using a student t-test. Specific analyses are described in more detail in the figure legend. Remaining analysis involved the use of a one-way ANOVA, followed by a Tukey post-hoc test to identify differences unless otherwise stated. Statistically significant differences are defined as *p < 0.05.

## Supplementary Information

**Additional file 1:** Higher doses of sodium valproate (valproate) affect morphology of transgenic MJD zebrafish. **A** Brightfield images of 6 day old i) non-transgenic, ii) EGFP-Ataxin-3 23Q, iii) EGFP-Ataxin-3 84Q vehicle, iv) 3.125 µM valproate treated, v) normal 6.25 µM valproate EGFP-Ataxin-3 84Q and vi) abnormal 6.25 µM valproate treated EGFP-Ataxin-3 84Q zebrafish larvae. Scale bar represents 500 µm. **B** Percentage of normal morphology reveals 6.25 µM valproate treated EGFP-Ataxin-3 84Q larvae had decreased normal morphology (****p < 0.0001; n = 6). **C** Non-transgenic zebrafish treated with 3.125 µM valproate between 1 and 6 days of age resulted in no changes to the distance swum compared to the vehicle treated control (*p* = 0.546; n = 68–70). Data represents mean ± SEM. Comparisons of the percentage of normal morphology were analysed using a one-way ANOVA followed by a Tukey post-hoc analysis and swimming distance was analysed using an unpaired t-test.

**Additional file 2:** Table of proteins identified in MJD transgenic zebrafish from mass spectrometry.

**Additional file 3:** Using label-free quantitative proteomics results, IPA demonstrated clustering of components of EIF signalling and the ubiquitin-proteome system and predicted the inactivation of the EIF2 signalling pathway. Green indicates downregulation (0.67-fold) and red indicates upregulation (1.5-fold) of proteins in valproate treated EGFP-Ataxin-3 84Q zebrafish compared to the vehicle controls.

**Additional file 4:** IPA predicted inhibition of biological and disease functions **A** “Degeneration of neurons” (Z-score − 1.062 p-value = 3.3 × 10–4) and **B** “Apoptosis” (Z-score − 1.246 *p*-value = 2.03 × 10–5) suggesting cell death processes were suppressed upon valproate treatment. Green indicates downregulation (0.67-fold) and red indicates upregulation (1.5-fold) of proteins in valproate treated EGFP-Ataxin-3 84Q zebrafish compared to the vehicle controls. Blue indicates predicted inhibition and orange indicates predicted activation of categorised biological function/pathway.

**Additional file 5:** Cells expressing polyglutamine expanded human ataxin-3 have decreased levels of SIRT1 protein. **A** Immunoblot of HEK293 cells stably expressing an empty vector, ataxin-3 28Q and ataxin-3 84Q probed for human ataxin-3 and SIRT1. All of the cells, including those expressing just the empty vector, carried the endogenous human ataxin-3 band, which ran at a similar height to the human ataxin-3 28Q band. Cells expressing human ataxin-3 84Q showed decreased SIRT1 levels. **B** Densitometric analysis of SIRT1 levels confirmed decreased levels of SIRT1 in the polyQ expanded ataxin-3 cells compared to the ataxin-3 23Q cells (*p* = 0.0213, n = 8–9 independent experiments). Data represents mean ± SEM. Statistical analysis performed was a one-way ANOVA followed by a Tukey post-hoc analysis.

**Additional file 6:** Resveratrol increases the swimming capacity of non-transgenic zebrafish at 6 days of age. Treatment of non-transgenic zebrafish with resveratrol (50 µM) from between 1 and 6 days of age produced an increase in total swimming distance compared to the vehicle treated control (**p = 0.005; n = 67–72). Data represents mean ± SEM. Statistical analysis performed was an unpaired t-test.

## Data Availability

The dataset supporting the conclusions of this article is included within the article (and its additional files), as well as within the PRIDE partner repository with the dataset identifier PXD009612 (Username: reviewer27566@ebi.ac.uk; Password: ta6siSAA).
