# Supplementary figures and images for: Sodium valproate increases activity of the sirtuin pathway resulting in beneficial effects for spinocerebellar ataxia-3 in vivo
Source: Mol Brain. 2021 Aug 20;14:128. doi: 10.1186/s13041-021-00839-x (PMC8377983; doi:10.1186/s13041-021-00839-x)

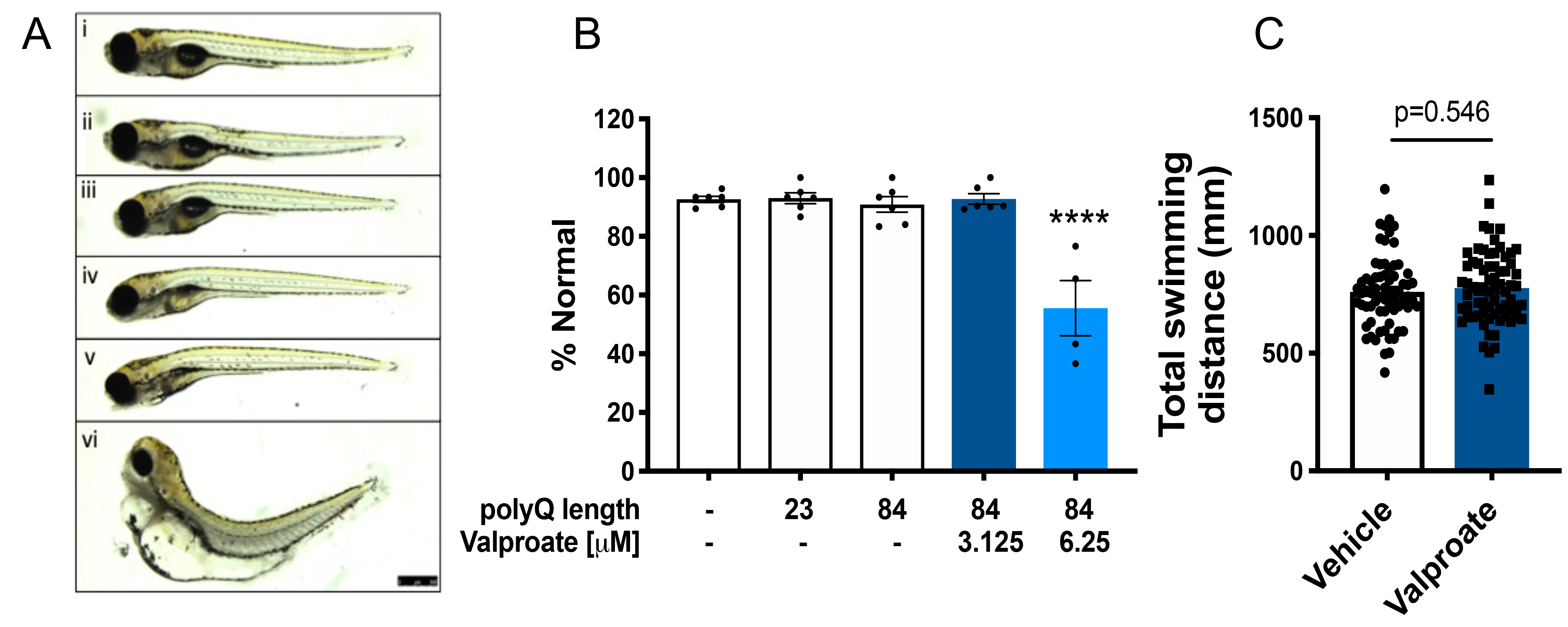

Supplement: Supplementary file 1 — Additional file 1: Higher doses of sodium valproate (valproate) affect morphology of transgenic MJD zebrafish. A Brightfield images of 6 day old i) non-transgenic, ii) EGFP-Ataxin-3 23Q, iii) EGFP-Ataxin-3 84Q vehicle, iv) 3.125 µM valproate treated, v) normal 6.25 µM valproate EGFP-Ataxin-3 84Q and vi) abnormal 6.25 µM valproate treated EGFP-Ataxin-3 84Q zebrafish larvae. Scale bar represents 500 µm. B Percentage of normal morphology reveals 6.25 µM valproate treated EGFP-Ataxin-3 84Q larvae had decreased normal morphology (****p < 0.0001; n = 6). C Non-transgenic zebrafish treated with 3.125 µM valproate between 1 and 6 days of age resulted in no changes to the distance swum compared to the vehicle treated control (p = 0.546; n = 68–70). Data represents mean ± SEM. Comparisons of the percentage of normal morphology were analysed using a one-way ANOVA followed by a Tukey post-hoc analysis and swimming distance was analysed using an unpaired t-test. [file 13041_2021_839_MOESM1_ESM.tif]

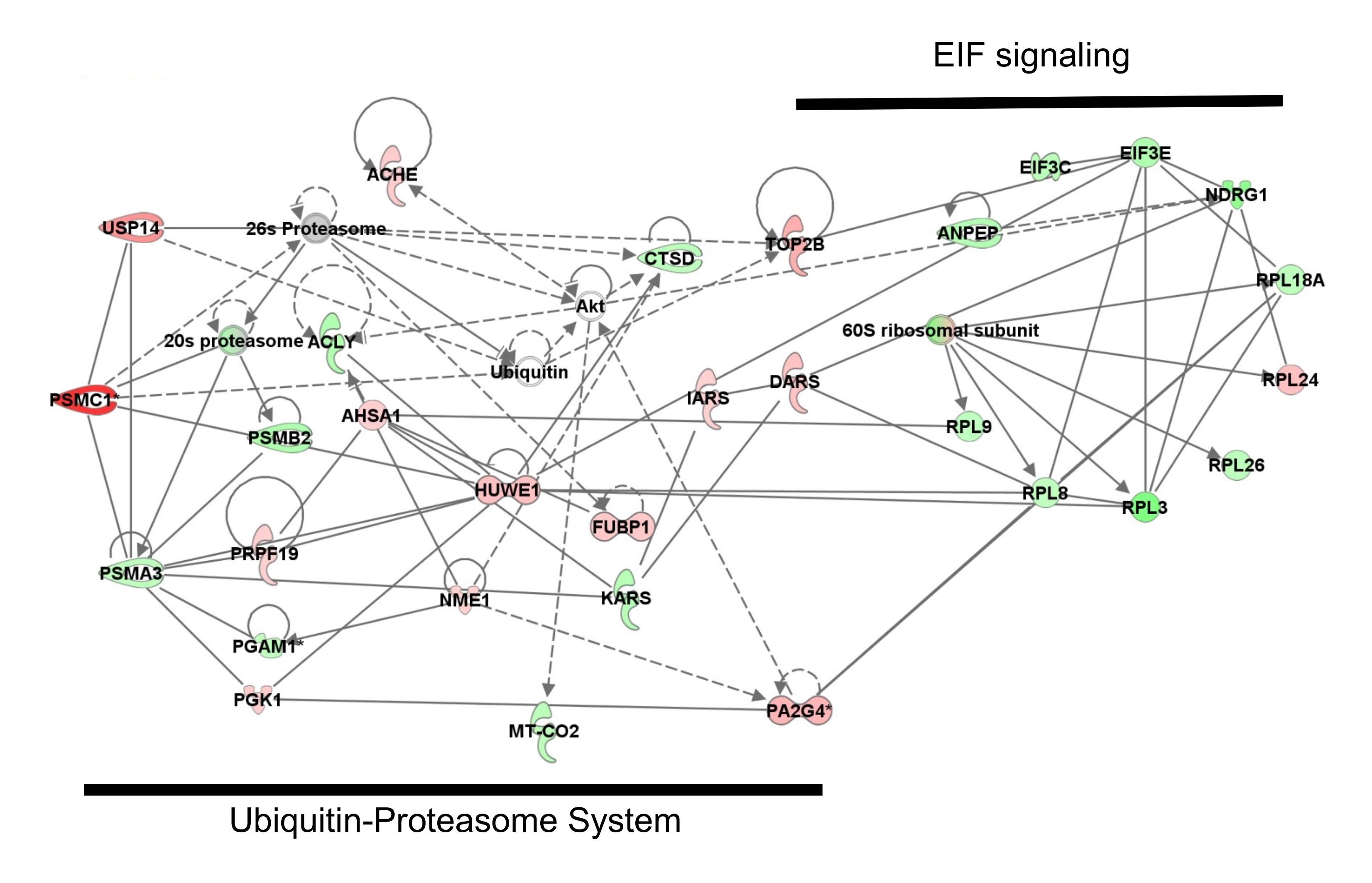

Supplement: Supplementary file 3 — Additional file 3: Using label-free quantitative proteomics results, IPA demonstrated clustering of components of EIF signalling and the ubiquitin-proteome system and predicted the inactivation of the EIF2 signalling pathway. Green indicates downregulation (0.67-fold) and red indicates upregulation (1.5-fold) of proteins in valproate treated EGFP-Ataxin-3 84Q zebrafish compared to the vehicle controls. [file 13041_2021_839_MOESM3_ESM.tif]

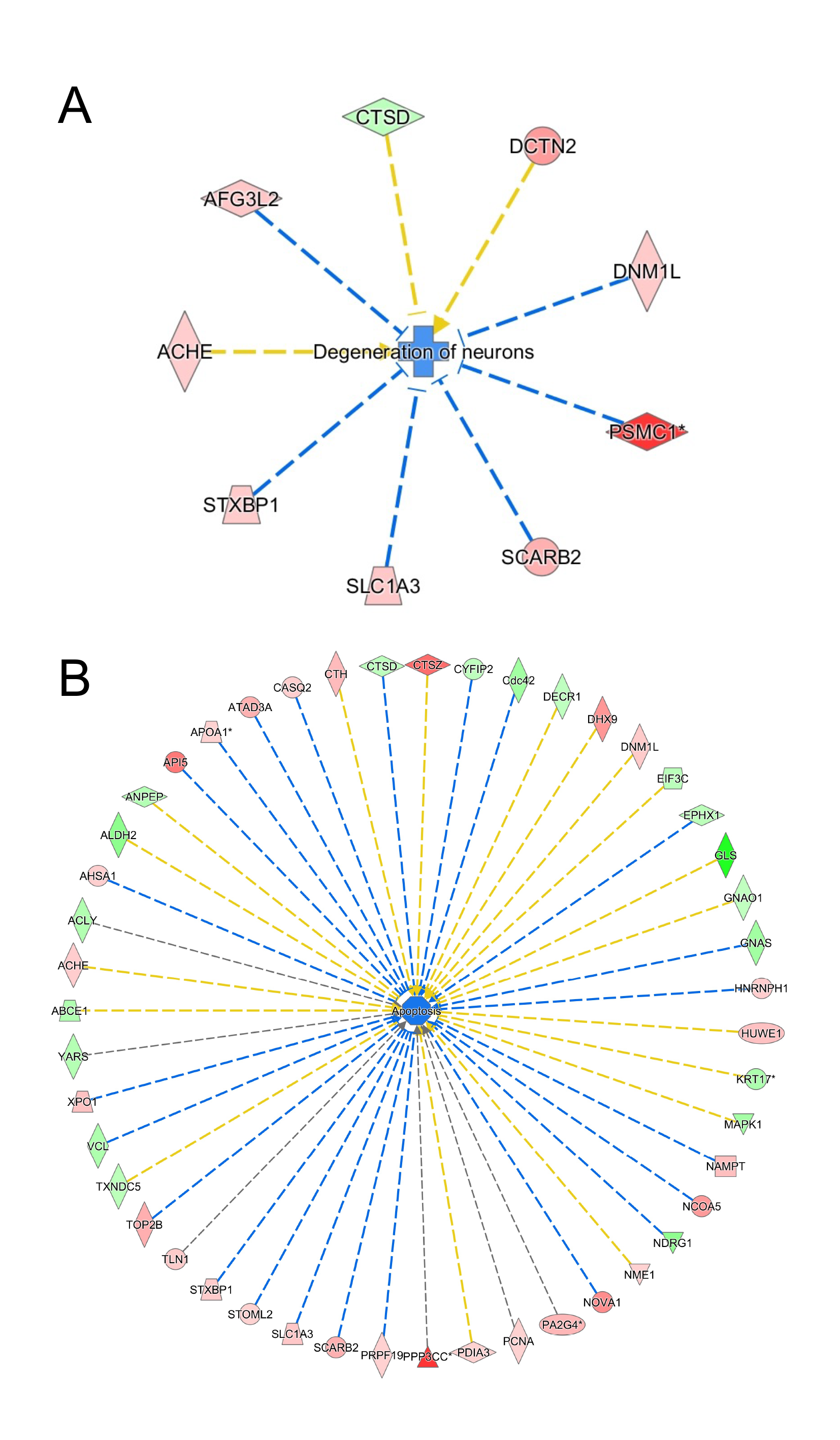

Supplement: Supplementary file 4 — Additional file 4: IPA predicted inhibition of biological and disease functions A “Degeneration of neurons” (Z-score − 1.062 p-value = 3.3 × 10^–4^) and B “Apoptosis” (Z-score − 1.246 p-value = 2.03 × 10^–5^) suggesting cell death processes were suppressed upon valproate treatment. Green indicates downregulation (0.67-fold) and red indicates upregulation (1.5-fold) of proteins in valproate treated EGFP-Ataxin-3 84Q zebrafish compared to the vehicle controls. Blue indicates predicted inhibition and orange indicates predicted activation of categorised biological function/pathway. [file 13041_2021_839_MOESM4_ESM.tif]

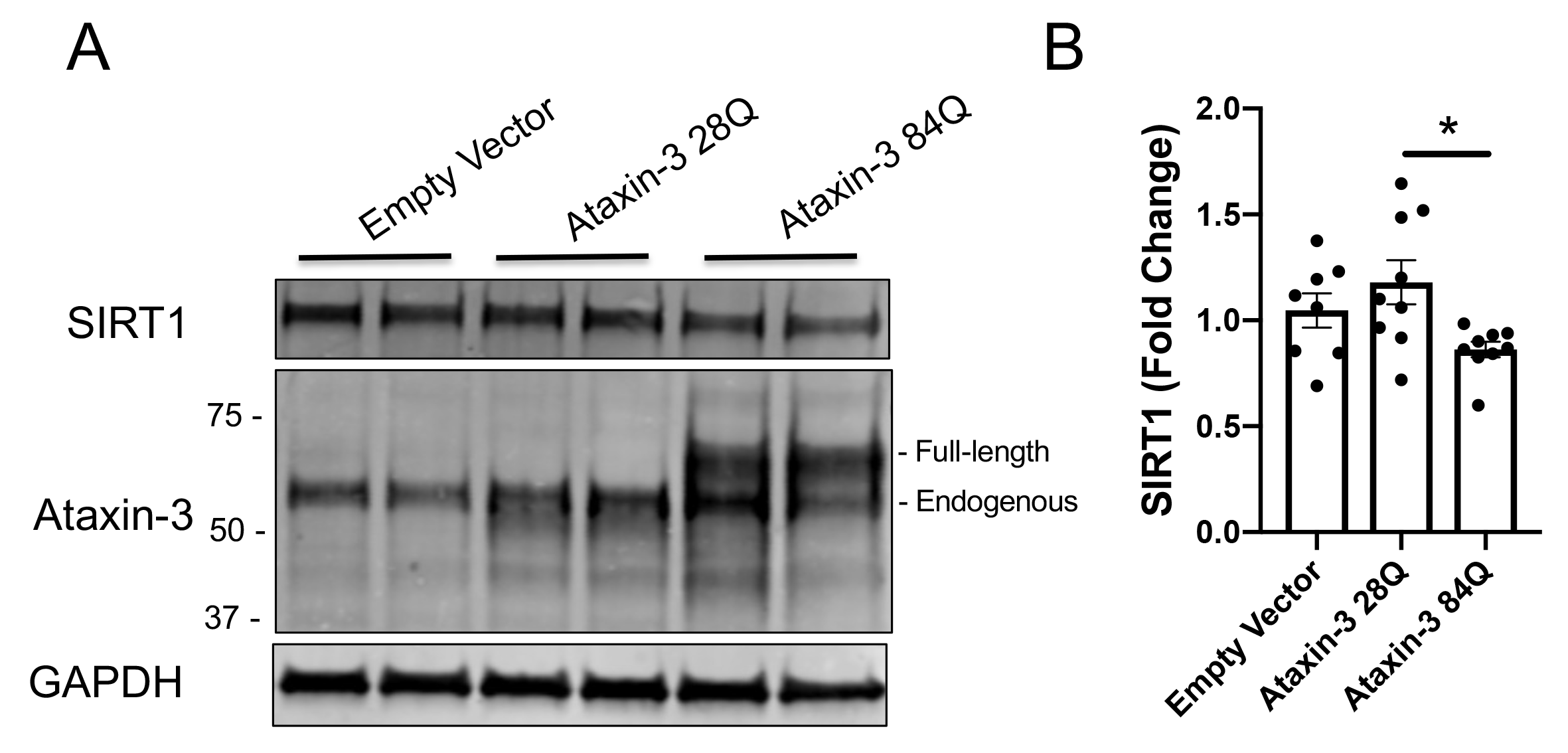

Supplement: Supplementary file 5 — Additional file 5: Cells expressing polyglutamine expanded human ataxin-3 have decreased levels of SIRT1 protein. A Immunoblot of HEK293 cells stably expressing an empty vector, ataxin-3 28Q and ataxin-3 84Q probed for human ataxin-3 and SIRT1. All of the cells, including those expressing just the empty vector, carried the endogenous human ataxin-3 band, which ran at a similar height to the human ataxin-3 28Q band. Cells expressing human ataxin-3 84Q showed decreased SIRT1 levels. B Densitometric analysis of SIRT1 levels confirmed decreased levels of SIRT1 in the polyQ expanded ataxin-3 cells compared to the ataxin-3 23Q cells (p = 0.0213, n = 8–9 independent experiments). Data represents mean ± SEM. Statistical analysis performed was a one-way ANOVA followed by a Tukey post-hoc analysis. [file 13041_2021_839_MOESM5_ESM.tif]

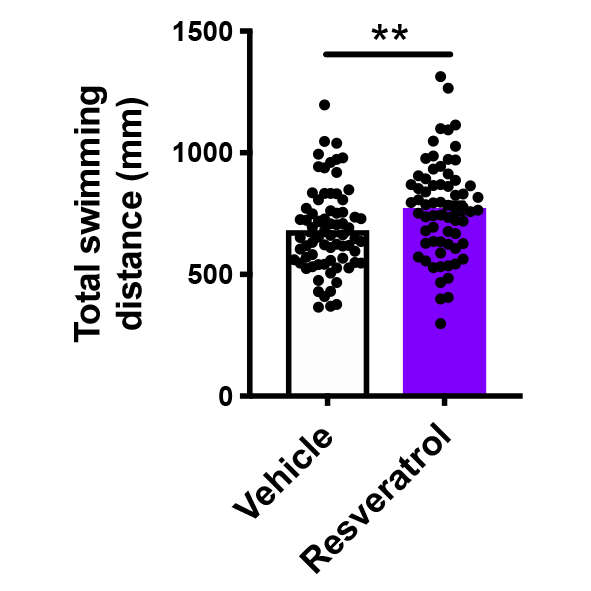

Supplement: Supplementary file 6 — Additional file 6: Resveratrol increases the swimming capacity of non-transgenic zebrafish at 6 days of age. Treatment of non-transgenic zebrafish with resveratrol (50 µM) from between 1 and 6 days of age produced an increase in total swimming distance compared to the vehicle treated control (**p = 0.005; n = 67–72). Data represents mean ± SEM. Statistical analysis performed was an unpaired t-test. [file 13041_2021_839_MOESM6_ESM.tif]
